# Comparative genome analysis reveals key genetic factors associated with probiotic property in *Enterococcus faecium* strains

**DOI:** 10.1186/s12864-018-5043-9

**Published:** 2018-09-04

**Authors:** Vikas C. Ghattargi, Meghana A. Gaikwad, Bharati S. Meti, Yogesh S. Nimonkar, Kunal Dixit, Om Prakash, Yogesh S. Shouche, Shrikant P. Pawar, Dhiraj P. Dhotre

**Affiliations:** 1 National Centre for Microbial Resource (NCMR), National Centre for Cell Science (NCCS), Pune, Maharashtra 411021 India; 2 Department of Biotechnology, Basaveshwar Engineering College, Bagalkot, Karnataka 587102 India

**Keywords:** Non-pathogenic, Pathogenic, Indian, Comparative genome analysis, *In-silico* analysis

## Abstract

**Background:**

*Enterococcus faecium* though commensal in the human gut, few strains provide a beneficial effect to humans as probiotics while few are responsible for the nosocomial infection. Comparative genomics of *E. faecium* can decipher the genomic differences responsible for probiotic, pathogenic and non-pathogenic properties. In this study, we compared *E. faecium* strain 17OM39 with a marketed probiotic, non-pathogenic non-probiotic (NPNP) and pathogenic strains.

**Results:**

*E. faecium* 17OM39 was found to be closely related with marketed probiotic strain T110 based on core genome analysis. Strain 17OM39 was devoid of known vancomycin, tetracycline resistance and functional virulence genes. Moreover, *E. faecium* 17OM39 genome was found to be more stable due to the absence of frequently found transposable elements. Genes imparting beneficial functional properties were observed to be present in marketed probiotic T110 and 17OM39 strains. Genes associated with colonization and survival within gastrointestinal tract was also detected across all the strains.

**Conclusions:**

Beyond shared genetic features; this study particularly identified genes that are responsible for imparting probiotic, non-pathogenic and pathogenic features to the strains of *E. faecium*. Higher genomic stability, absence of known virulence factors and antibiotic resistance genes and close genomic relatedness with marketed probiotics makes *E. faecium* 17OM39 a potential probiotic candidate. The work presented here demonstrates that comparative genome analyses can be applied to large numbers of genomes, to find potential probiotic candidates.

**Electronic supplementary material:**

The online version of this article (10.1186/s12864-018-5043-9) contains supplementary material, which is available to authorized users.

## Background

Probiotic organisms according to World Health Organisation are ‘Live microorganisms which when administered in adequate amounts confer a health benefit on the host’. Beneficial effects may include genes/pathways for production of vitamins, essential amino acids, antioxidants, digestion of complex carbohydrates, susceptibility to antibiotics, antagonism against enteric bacteria and modulation of immune system. But along with conferring health benefits the organism should have or lack series of properties. The probiotic strain should have genes to compete, adhere, persist and survive in the harsh conditions of the gastrointestinal tract (GIT). Moreover, the probiotic strain should show absence of any virulence factors and multi drug resistance. Pathogenic bacteria may contain genes for survival in GIT along with some beneficial properties, but they hold virulence factors which help them to evade the host immune response and eventually cause disease. Additionally, presence of antibiotic resistance genes in pathogens makes the treatment difficult in disease conditions. Apart from these, many bacteria in the gut do not show either probiotic or pathogenic properties and hence can be termed as non-pathogenic non-probiotic (NPNP).

The genus *Enterococcus* is one of the diverse and ecologically significant group, and members of this genus are ubiquitously distributed in nature viz. animals, human gastrointestinal tract (GIT) and plants [1–7]. *Enterococcus* plays an important role in the ripening of cheese products by lipolysis and proteolytic properties leading to the development of aroma and flavour [7]. In the Mediterranean region, *Enterococcus* spp. have been used in the preparation of various meat and fermented milk products for centuries [5]. Further, they also exhibit the beneficial property of bacteriocin production [5, 7] presenting activity against potential pathogens viz. group D streptococci and *Listeria* in various foods and GIT [7].

*E. faecium* is widely and extensively studied for its leading cause of nosocomial infections in humans [8]*.* It is a gut commensal and acts as an opportunistic pathogen due to a variety of virulence factors, including lipopolysaccharides and biofilm formation [9]. Their pathogenic nature is evident in urinary tract infections, endocarditis, and surgical wound infection, displaying its capability of causing a wide range of infections [10]. Another remarkable character of *E. faecium* is its tolerance to many antimicrobial drugs [11, 12]. It has also acquired the antibiotic-resistance gene against vancomycin and a multidrug resistance beta-lactamase gene [13]. Besides, it has been shown that *E. faecium* is capable of acquiring resistance to antibiotics by sporadic mutations and infections caused by these are normally difficult to treat [14]. The strains like Aus0004 and V583 are reported as pathogens [1].

Numerous studies in the last decade have validated the safety claim of *Enterococci* in foods and as probiotics [15–18]. The application of *Enterococci* as a starter culture e.g. *E. faecium* SF68 (Switzerland) and as probiotic e.g. *E. faecium* T110 (Japan) has been used widely [19, 20]. Additionally, *E. faecium* T110 is used in many commercially available probiotics, and no cause of illness or death has been reported [9]. *E. faecium* is among one of the directly fed microorganism recognized by the Association of American Feed Control, 2016. It is permitted as a probiotic supplement in the diet for poultry, dogs, piglets and mice [21–24]. Few strains of *E. faecium* (NRRL B-2354) act as surrogate microorganism used in place of pathogens for validation of thermal processing technologies [25] and some are widely used as laboratory strains, e.g. *E. faecium* 64/3 [26]. These two strains are non-pathogenic and are used routinely without any known disease outbreak [27]. Thus, the diversity and genomic plasticity of *E. faecium* are accountable for both probiotic and pathogenic nature [28–30]. In this study, we have carried out comparative genome analysis to identify genes/pathways which can help in distinguishing probiotic, pathogenic and NPNP strains of *E. faecium.* Further, we have also tried to describe the genetic differences between strain 17OM39 with marketed probiotic, non-pathogenic non-probiotic (NPNP), and pathogenic strains.

## Results

### Strain selection

Whole genome sequences were downloaded from NCBI genome database, and the strains were grouped into probiotic, non-pathogenic non-probiotic (NPNP) and pathogenic based on the literature survey (Table 1). The pathogenic group had six strains: DO, Aus0004, Aus0085, 6E6, E39 and ATCC 700221 [1–4]. The first four were isolated from the human blood and later two from human stool. The NPNP group had two strains: NRRL B-2354 and 64/3 [25, 26]. The probiotic group had the marketed strain T110 [9] and strain 17OM39 isolated from healthy human gut [31].

**Table 1:** General Genome Features

Features	Probiotic		Non-Pathogenic		Pathogenic					
	T110	17OM39	NRRLB-2354	64/3	DO	Aus0004	Aus0085	6E6	E39	ATCC 700221
Size (mb)	2.6	2.6	2.6	2.5	2.6	2.9	2.9	2.9	2.7	2.8
GC%	38.4	38.5	37.8	38.2	37.9	38.3	37.9	37.6	37.8	37.8
Genes	2639	2865	2771	2508	2795	2960	3214	3404	3043	3145
CDS	2502	2639	2658	2418	2703	2825	2938	3307	2907	2725
Pseudo Genes	54	148	47	32	32	70	181	73	44	326
rRNAs (5S, 16S, 23S)	6, 6, 6	6, 6,6	6, 6, 6	6, 6, 6	6, 6, 6	6, 6, 6	6, 6, 6	6, 6, 6	6, 6, 6	6, 6, 6
tRNAs	65	62	48	68	62	47	76	75	70	72
Plasmid	1	–	1	–	3	3	6	2	5	3
Accession code	NZ_CP006030.1	LWHF 00000000.1	NC_020207.1	NZ_CP012522.1	NC_017960.1	NC_017022.1	NC_021994.1	NZ_CP013994.1	NZ_CP011281.1	CP 014449.1
Source	probiotic	feces	cheese	feces	blood	blood	blood	feces	blood	feces
Country	Japan	India	–	Germany	USA	Australia	Australia	USA	USA	USA
Reference	Natarajan and Parani 2015	Ghattargi et al. 2018	Kopit et al. 2014	Bender et al. 2015	Lam et al. 2012	Qin et al. 2012	Qin et al. 2012	Geldart and Kaznessis 2017	Geldart and Kaznessis 2017	McKenney et al. 2016

### General genomic features

Genome sizes ranged from approximately 2.57–2.99 Mb with strain DO exhibiting the smallest and 6E6 the largest genome. Average G + C content varied between 37.25 to 38.55%. The genomic features of strains under the study are provided in Table 1. No significant differences (*p*-value ≤0.05, Kruskal–Wallis test) could be noted between the groups with respect to their genome size, G + C content, average number of genes and coding DNA sequences (CDS).

The RAST annotation has facilitated to determine the features assigned to subsystems that are present in all organisms (Additional file 1: Figure S1). The average numbers of annotated protein-encoding genes were 2570, 2639 and 3093 for probiotic, NPNP and pathogenic groups, respectively. Annotation based on RAST for strains under the study suggested high abundance of subsystems related to carbohydrates and protein metabolism.

### Comparisons of 17OM39 with other *E. faecium* strains

**Fig. 1:**
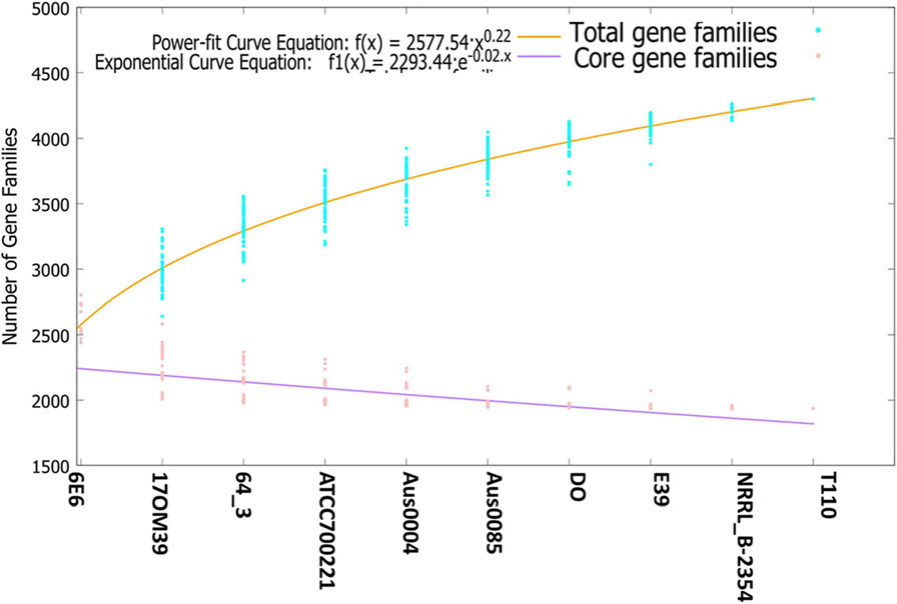
Core and pan genome of *E. faecium* strains. The number of shared genes was plotted as a function of number of strains (n) added sequentially. 1935 genes were shared by all 10 genomes. The orange line represents the least-squares fit to the power law function f(x) = a.x^b where a = 2577.54, b = 0.222602. The red line represents the least-squares fit to the exponential decay function f1(x) = c.e^(d.x) where c = 2293.44, d = − 0.0232013

The availability of nearly complete *E. faecium* genomes has helped to define the core, accessory and unique genomic features for all the strains. The comparison of strain 17OM39 with other strains of probiotic, NPNP and pathogenic strains, revealed 1935 (85.53%) core genes, 526 (20.64%) accessory and 87(3.41%) unique genes. The numbers of shared genes were plotted as a function of number of strains (Fig. 1).

### Pan-genome analysis

**Fig. 2:**
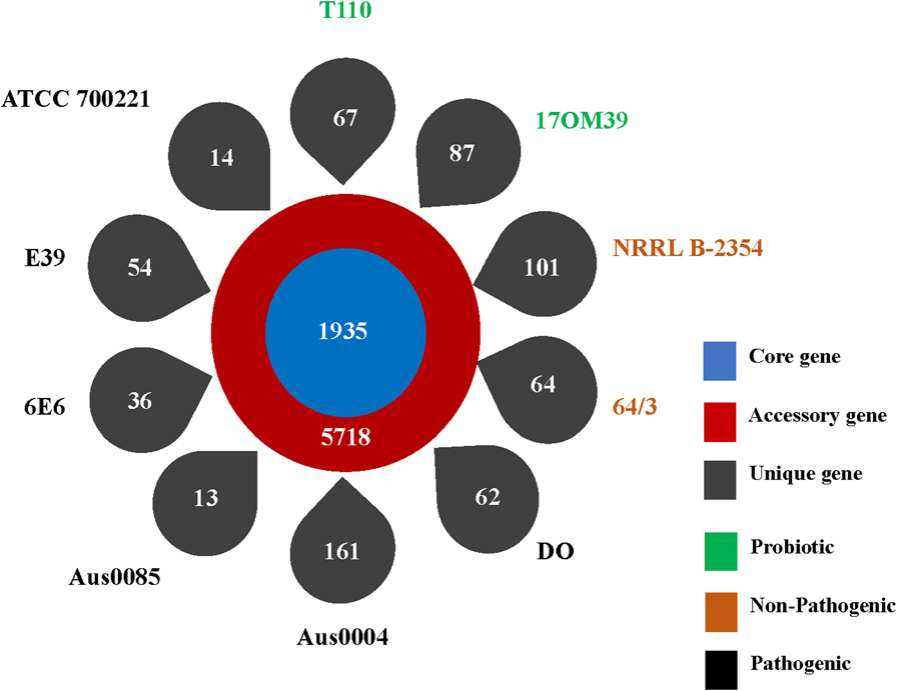
Number of core, accessory and unique gene families of *E. faecium* genomes. The inner circle represents core genome consisting of 1935 genes. The outer red circle represents accessory genomes for all ten strains adding to a sum of 5718 genes, while the outer petals represent unique genes associated with all the strains. Green color indicate probiotic strains, brown are NPNP and black are pathogenic

The pan-genome analysis revealed presence of 1935 core genes and 5718 accessory genes (Fig. 2). The numbers of strain-specific genes observed were 67, 87, 10, 64, 62, 16, 13, 36, 54 and 14 for strains 17OM39, T110, NRRL B-2354, 64/3, DO, AUS0004, AUS0085, 6E6, E39 and ATCC700221, respectively (Fig. 2). Identification of core, accessory and unique gene families by orthoMCL analysis revealed the proportion of known, hypothetical and uncharacterized proteins in these groups (Additional file 1: Figure S2A). Large percentages (61.19%) of unique genes in all genomes were assigned to an uncharacterized group, and further studies will be required to examine the unexplored attributes.

### Core genome analysis

orthoMCL analysis of core genes led to the identification of 850 genes present in single copy and 772 genes present in multiple copies in all ten strains. Functional analysis of core genes showed distribution in a varied range of functional categories within Cluster of Orthologous Genes (COG) viz. growth, DNA replication, transcription, translation, carbohydrate and amino acid metabolism, stress response and transporters. Categories representing transport and metabolism of coenzymes, lipids, amino acids and nucleotides comprised of 16.24% of the core genes, while 11.30% of core genes were ascribed to carbohydrate metabolism.

### Accessory genome analysis

**Fig. 3:**
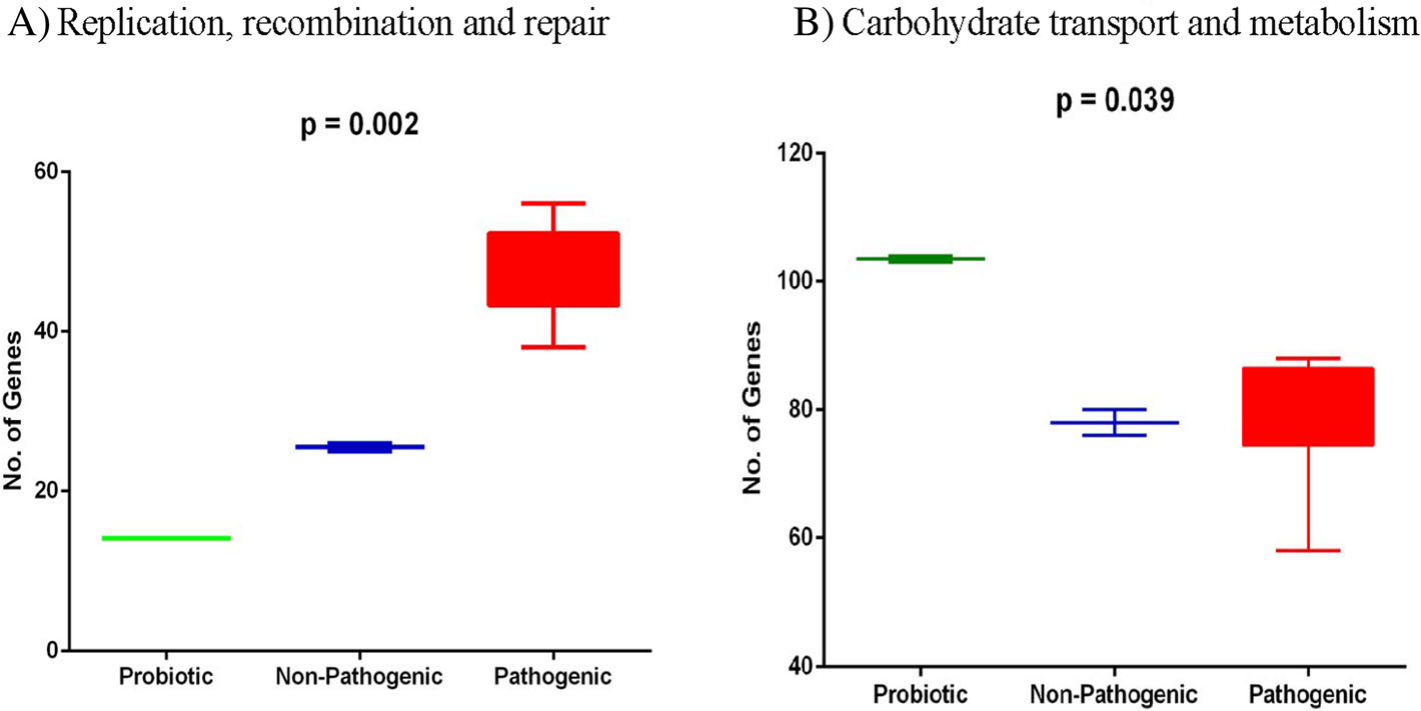
Showing the significant COG’s in accessory genome. **a** Replication, recombination and repair (**b**) Carbohydrate transport and metabolism

Functional analysis of the accessory genes showed diverse distribution in COG categories as similar to core gene annotations (Additional file 1: Figure S3). Two important subsystems observed in accessory genes were a) carbohydrate metabolism and b) replication, recombination and repair systems. The former was abundant in the probiotic group (*p* = 0.002) while later in the pathogenic group (*p* = 0.039) (Fig. 3). We also attempted to find accessory genes shared between the groups. The probiotic and pathogenic group shared 15 genes; four of them were general transporters, two were manganese-containing catalase gene while others were hypothetical proteins (Additional file 1: Figure S2B).

### Unique genome analysis

The important unique genes associated with various strains were as follows: Phosphotransferase (PTS) system for mannose/fructose/sorbose was found in probiotic strain 17OM39. Marketed probiotic strain T110 had macrolide-efflux transmembrane protein which acts as a drug efflux pump. The important unique genes for other strains were hexosyltransferase in strain 64/3, type III restriction-modification system in strain NRRL B- 2354, Cro/CI family transcriptional regulator protein in strain 6E6, transposase for insertion sequence IS1661 in strain ATCC 700221, streptogramin A acetyltransferase in strain Aus0004, Patatin-like proteins in strain Aus0085, IS1668 transposase in strain DO and plasmid recombination enzyme in strain E39. Additional file gives detailed information on core, accessory and unique genes (see Additional files 2, 3, 4).

### Antibiotic resistance determinants

Screening of antibiotic resistance determinants in genomes was necessary to understand if probiotic strains harboured these genes. Genes conferring resistance to kanamycin were found in all the genomes. The NPNP group showed presence of general multidrug transporter. Within pathogenic strains, Aus004 and Aus0085 showed presence of tetracycline, trimethoprim and vancomycin resistance gene. Strains E39 and 6E6 showed presence of genes responsible for trimethoprim and tetracycline resistance. Pathogenic strain E39 presented daptomycin resistance gene and strain ATCC 700221 showed presence of genes responsible for resistance to the antimicrobial activity of cationic antimicrobial peptides and antibiotics such as polymyxin. Table 2 shows presence of various antibiotic resistance genes found in each strain.

**Table 2:** Antibiotic Resistance genes found in *Enterococcus* genomes as performed by CARD analysis, where + Present and - Absent

Property	Probiotic		Non-pathogenic Non-Probiotic		Pathogenic					
Antibiotics	T110	17OM39	NRRL B-2354	64_3	DO	Aus0004	Aus0085	6_E6	E39	ATCC 700221
Daptomycin	–	–	–	–	–	–	–	–	+	–
Trimethoprim	–	–	–	–	–	+	+	+	+	–
Multidrug	–	–	–	–	–	+	–	–	–	–
Macrolide	–	–	–	–	–	+	–	–	–	–
Polymyxin	–	–	–	–	–	–	–	–	–	+
Tetracyline	–	–	–	–	–	+	+	+	+	–
Vancomycin	–	–	–	–	–	+	+	–	–	–

### Virulence determinants

Various virulence determinants such as adherence, biofilm formation and exo-enzyme production in probiotic, NPNP and pathogenic groups were identified. Genes (*acm, scm, Ebp*A*, Ebp*C) described as adherence factors have been attributed to pathogenic effects. Excluding strain DO all other pathogenic strains showed presence of enterococcus surface protein (*esp)* gene. The *bop*D gene involved in biofilm was intact in all groups, but the operon was absent in strain 17OM39, marketed probiotic strain T110 and NPNP strains. In an exo-enzyme group, hyaluronidase gene was found to be associated with marketed probiotic strain alone, while the gene in strain 17OM39 displayed an alteration in sequence at position 167 (G > T) suggesting this could affect its functionality due to the nonsense mutation. Gene *acm* in the probiotic and the non-pathogenic group was not functional due to the non-sense mutation at position 1060 (G > T). Also, virulence genes *scm, efa*A and *srt*C are not well characterized as virulence determinants in *E. faecium* [9] (Table 3).

**Table 3:** Virulence factors found in *Enterococcus* genomes, where + Present; − Absent; * Non-functional due to presence of stop codon

Property		Probiotic		Non-pathogenic Non-probiotic		Pathogenic					
CATEGORY	GENES	T110	17OM39	NRRL B-2354	64/3	DO	Aus004	Aus0085	6E6	E39	ATCC 700221
Adherence	*acm*	*	*	*	*	**+**	**+**	**+**	**+**	**+**	**+**
	*EbpA*	**–**	**–**	**+**	**+**	**+**	**+**	**+**	**+**	**+**	**+**
	*EbpC*	**–**	**–**	**+**	**+**	**+**	**+**	**+**	**+**	**+**	**+**
	*srtC*	**+**	**+**	**+**	**+**	**+**	**+**	**+**	**+**	**+**	**+**
	*EcbA*	**–**	**–**	**–**	**–**	**+**	**+**	**+**	**+**	**+**	**+**
	*efaA*	**+**	**+**	**+**	**+**	**+**	**+**	**+**	**+**	**+**	**+**
	*Esp*	**–**	**–**	**–**	**–**	**–**	**+**	**+**	**+**	**+**	**+**
	*Scm*	*	*	*	*	**+**	**+**	**+**	**+**	**+**	**+**
	*SgrA*	**–**	**–**	**–**	**–**	**+**	**+**	**+**	**+**	**+**	**+**
Bioflim	*bopD*	*	*	*	*	**+**	**+**	**+**	**+**	**+**	**+**
Exoenzymes	*EF0818*	**+**	*	**–**	**–**	**–**	**–**	**–**	**–**	**–**	**–**

### Mobile genetic elements

**Fig. 4:**
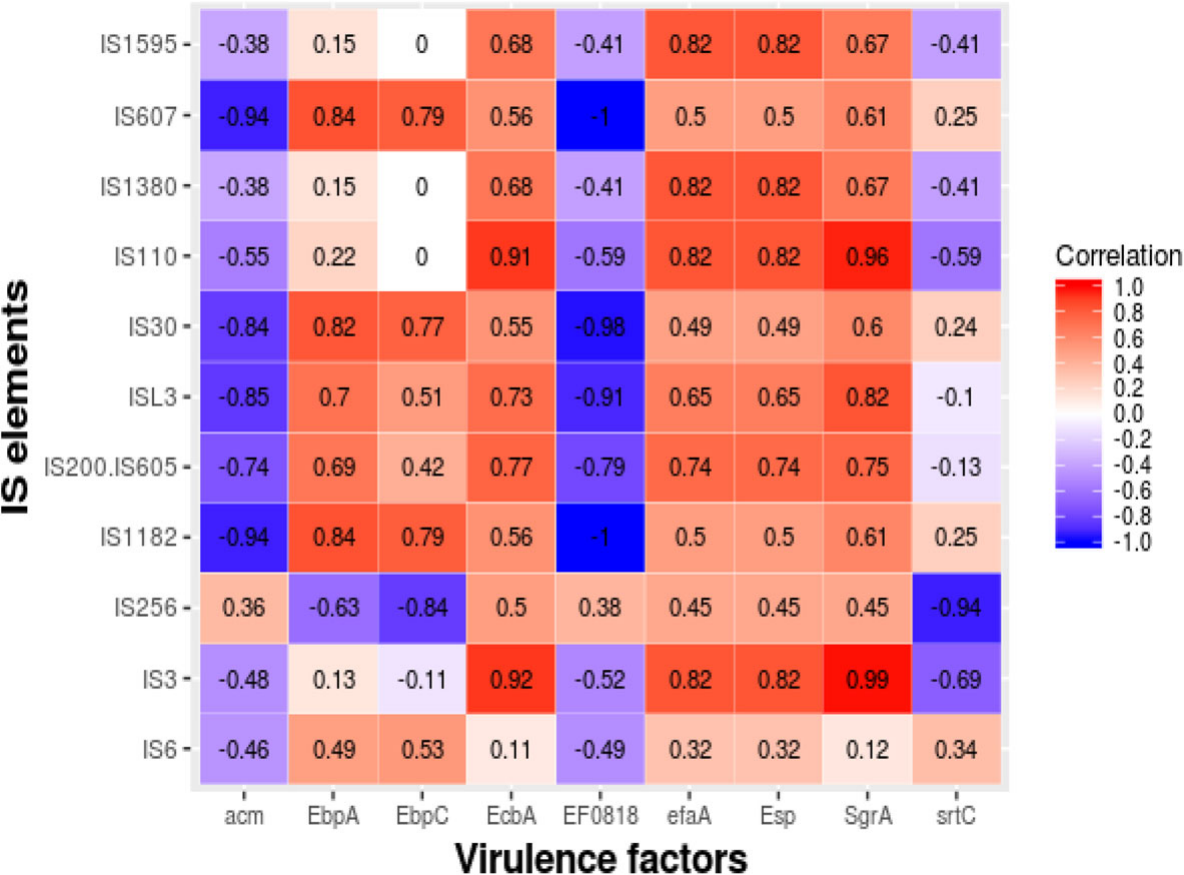
Heatmap showing correlation between IS elements and virulence factors found across the genomes. Red color indicated strong positive correlation while blue indicated negative correlation

A number of Mobile Genetic Elements (MGEs) have been described in *E. faecium* including transposons, plasmids, and bacteriophage [32]. Based on the screening for Insertion sequences (ISs) (Additional file 1: Table S1), the IS1542 was present only in probiotic strains. The IS element ISEfa12 was present only in NPNP group and IS1216, 1S1216E, 1S1216V, IS16, IS6770, ISEf1, ISEfa10, ISEfa11, ISEfa5, ISEfa7, ISEfa8, ISEnfa3, ISS1W were present only in the strains belonging to the pathogenic group. The detection of IS16 element was seen only in pathogenic group of *E. faecium* strains. A strong correlation between IS elements and virulence factors was observed in all genomes (Fig. 4) suggesting that these IS elements might have a role in transfer of these virulence factors. Further studies are required to verify these findings.

We could trace at-least one intact prophage in all ten genomes (Additional file 1: Table S2). In total, we could identify three incomplete (PHASTER score < 70) and twenty-three intact phages (PHASTER score < 70–90). Nine phages whose completeness status was doubtful (PHASTER score < 90) were also identified. The NPNP strains had two intact prophages, while the probiotic strain 17OM39 had one intact and strain T110 had two intact prophages. *E. faecium* ATCC 700221 had the highest number of intact phages (Additional file 1: Table S2). Further, Clustered Regularly Interspaced Short Palindromic Repeat (CRISPR) associated (Cas) system were found to be absent within the all genomes.

**Fig. 5:**
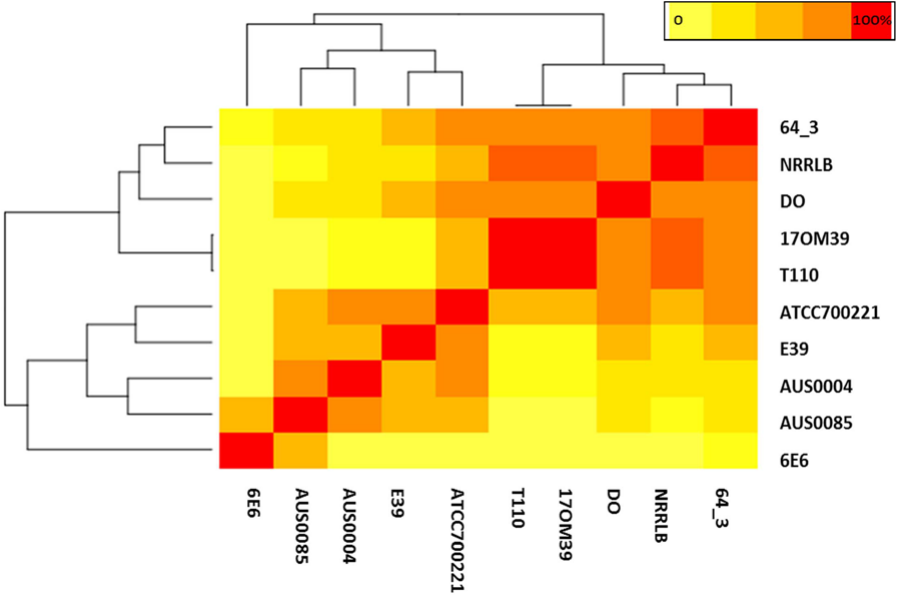
Heatmap showing similarities between genomic islands of the strains considered in this study. The light-yellow shows least percent similarity while the red indicate 100% similarity in all genomes. Pathogenic strains (E39, Aus0004, Aus0085 and 6E6) showed clustering while probiotic strains (T110 & 17OM39), NPNP strains (64_3 & NRRLB) and pathogenic strain DO show separate cluster. The color scheme is as shown in the legend

**Fig. 6:**
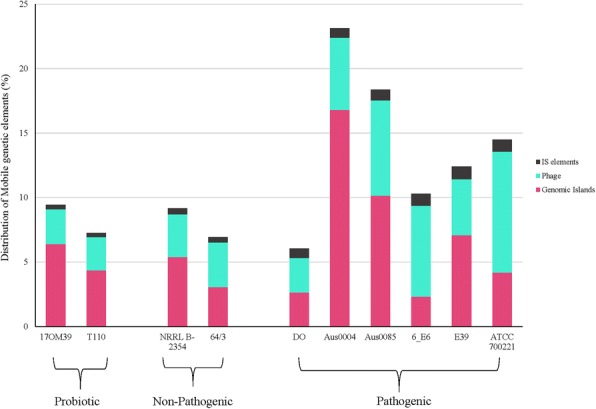
Proportion of mobile genetic elements across *Enterococcus* genomes. The pink colour shows the proportion of genomic islands present in each strain, light green for bacteriophages and black of IS elements in all the strains. Strain Aus004 has nearly quarter of its genome comprised of mobile genetic elements

Genomic islands of strain 17OM39 were compared with the other strain of *E. faecium* to find out genes transferred by Horizontal Gene Transfer (HGT) (Additional file 1: Table S3). We identified a total of 11 genomic islands in strain 17OM39 amounting to 3.5% of the total genome. The genomic island GI1 was common across all groups except for the strain 6E6. The choloylglycine hydrolase gene was found to be present in the genomic island of probiotic strain T110, pathogenic strain 6E6 and NPNP strain 64/3. Pathogenic group showed a large number of IS elements, transposons and antibiotic resistance genes within genomic island. All pathogenic strains showed presence of tetracycline resistance gene and cell adhesion protein within the genomic island. Only two pathogenic strains Aus0004 and Aus0085 showed presence of *esp* (enterococcal surface protein) virulence gene, and vancomycin resistance genes. A higher similarity was observed between genomic islands of probiotic and NPNP strains as compared to pathogenic group (Fig. 5). Pathogenic strain AUS0004 had the highest mobile genome with almost 25% of its genome falling within the MGEs (Fig. 6). Additional file gives detailed information on genes present in genomic islands (see Additional file 5).

### Survival in gastrointestinal tract

Biologically active microorganisms are usually required at the target site to induce a health benefits or pathogenic effect. To induce such effects it is necessary for the organism to survive and persist in the GIT. Thus, a list of genes encoding for survival and growth were first identified in the strain 17OM39 and then compared with other strains. We found Permease IIC component gene only in the probiotic group. All the groups showed presence of genes that impart resistance to acid, bile and could hydrolyse bile salt. Moreover, these strains were also able to adhere and grow in the GIT (Table 4).

**Table 4:** Number of genes responsible for survival in GI track within *Enterococcus* genomes

Property		Probiotic		Non-pathogenic Non-probiotic		Pathogenic					
CATEGORY	GENES	T110	17OM39	NRRL B-2354	64/3	DO	Aus0004	Aus0085	6_E6	E39	ATCC 700221
Acid resistance	*LBA0995*	3/5	3/5	3/5	3/5	4/5	4/5	5/5	4/5	4/5	3/5
	*LBA1524*										
	*LBA1272*										
	*gadC*										
	*rrp-1*										
Bile resistance	*LBA1430*	4/4	4/4	4/4	4/4	4/4	4/4	4/4	4/4	4/4	4/4
	*clpE*										
	*dps*										
	*LBA1429*										
Competitive	*copA*	3/3	3/3	2/3	2/3	2/3	2/3	2/3	2/3	2/3	2/3
	*met*										
	*pts14C*										
Adherence	*lsp*	2/3	2/3	3/3	3/3	3/3	3/3	3/3	3/3	3/3	3/3
	*FbpA*										
	*ispA*										
Persistence	*LJ1656*	4/4	4/4	4/4	4/4	4/4	4/4	4/4	4/4	4/4	4/4
	*msrB*										
	*LJ1654*										
	*clpC*										
Bile salt hydrolase	*bsh*	1/1	1/1	1/1	1/1	1/1	1/1	1/1	1/1	1/1	1/1
Growth	*treC*	1/1	1/1	1/1	1/1	1/1	1/1	1/1	1/1	1/1	1/1
Adaptation	*Lr1265*	1/2	1/2	1/2	1/2	1/2	2/2	2/2	2/2	2/2	2/2
	*Lr1584*										

### Probiotic properties

As stated earlier the strain 17OM39 and marketed probiotic strains T110 were devoid of any clinically relevant antibiotic resistance gene while all the strains were able to survive in GIT. Strains 17OM39 and T110 (marketed probiotic) showed presence of complete pathways for essential amino acid synthesis viz. valine, lysine, and methionine and vitamins such as folate and thiamine (Table 5). Genes responsible for antibacterial activity (bacteriocin) specific against *Listeria* were found. Genes for exopolysaccharide (EPS) and anti-oxidant production (hydro-peroxidases) were noted which in-turn help the probiotic strains to establish themselves in the gut. The NPNP group only had EPS gene cluster. On the other hand, complete pathways for amino acid and vitamin synthesis were absent in NPNP and pathogenic group. Thus, probiotic strains have pathways/genes imparting beneficial effects to human host unlike NPNP and pathogenic group.

**Table 5:** Probiotic properties found in *Enterococcus* genomes, where **+** Present and - Absent

Property	Probiotic		Non-pathogenic Non-probiotic		Pathogenic					
Properties	17OM39	T110	64/3	NRRL B-2354	DO	Aus 0004	Aus 0085	6_E6	E39	ATCC 700221
Anti-oxidant	+	+	–	–	–	–	–	–	–	–
Anti-bacterial	+	+	–	–	–	–	–	–	–	–
EPS	+	+	+	+	–	–	–	–	–	–
Amino-acid	valine, lysine, methionine	valine, lysine	–	–	–	–	–	–	–	–
Vitamins	Folate, Thiamine	Folate, Thiamine	–	–	–	–	–	–	–	–

### Plasmids

Plasmids comprise a substantial portion of the accessory genome and are accountable for antibiotic and virulence properties. Thus, an attempt was made to compare the plasmids of strains considered in this study with respect to their virulence factors, antibiotic resistance, phage regions and IS elements. Plasmid-encoded gene in marketed probiotic strain T110 showed 66% similarity to the cytolysin (*cyl*) gene. Strains in the pathogenic group (6E6, ATCC700221, Aus0085, and E39) showed presence of plasmid-encoded genes for vancomycin, streptothricin, erythromycin, gentamicin and kanamycin resistance (Additional file 1: Table S4). Surprisingly, no phage elements were associated with the probiotic strain T110, while the non-pathogenic strain (NRRL B-2354) and pathogenic strains (ATCC 700221, Aus0085, DO, and E39) harboured incomplete or complete prophages in the plasmids. The list of IS elements found in the plasmids is summarised in Additional file 1: Table S5.

### Comparison of probiotic, NPNP and pathogenic strains

**Fig. 7:**
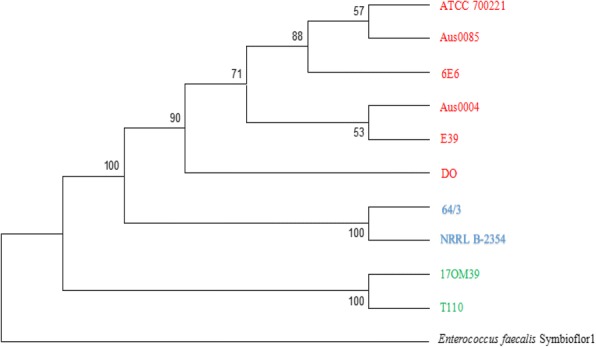
Core Genome Phylogeny. Phylogenetic tree of 10 *Enterococcus faecium* strains using the Maximum Likelihood method based on the GTR + G substitution model. The tree with the highest log likelihood (− 17,644.1414) is shown. Evolutionary analyses were conducted in MEGA6. A concatenated tree of core 1945 genes common in all the strains were considered in the final dataset

**Fig. 8:**
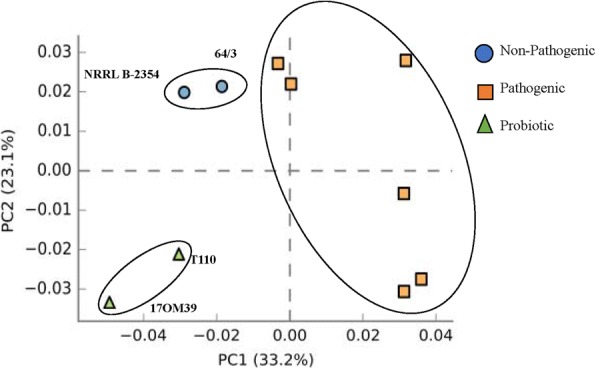
PCA plot comparing probiotic, pathogenic and NPNP *Enterococcus* genomes based on presence and absence of 67 genes responsible for survival in GI track, virulence factors and antibiotic resistance. The probiotic strains are shown in green, non-pathogenic in blue and pathogenic in red colour and clustering is indicated by oval shaped rings on the strains. From the plot, it can be noted that strain 17OM39 is different from the marketed probiotic strain T110

**Fig. 9:**
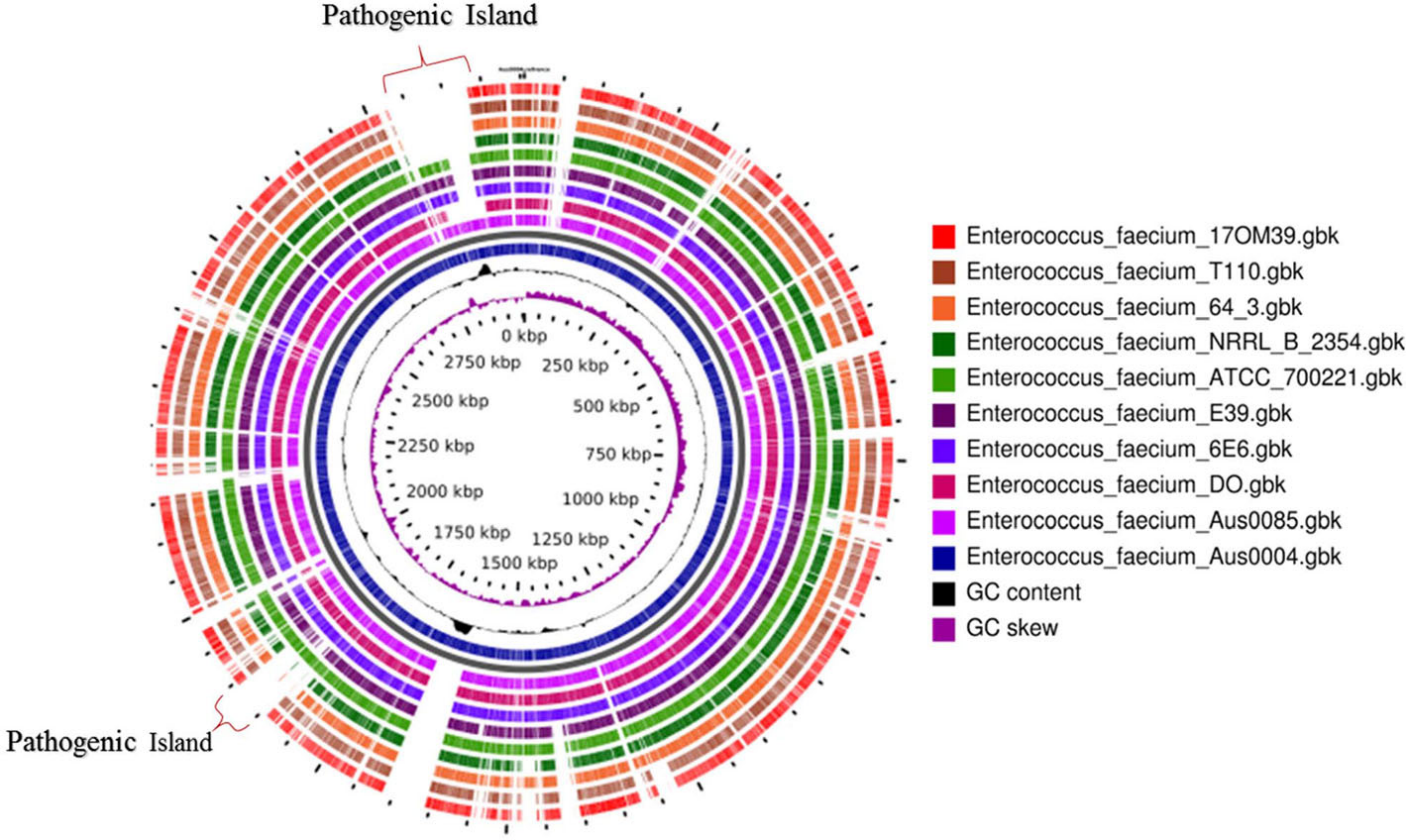
Blast Atlas of *Enterococcus* genomes, with strain Aus004 as a reference genome followed by Aus0085, DO, 6E_6, E_39, ATCC_7200221, NRRLB_2354, 64_3, T110 and the outermost as 17OM39. The two pathogenic islands (has most of virulence factors and antibiotic resistance genes) are shown in figure

**Fig. 10:**
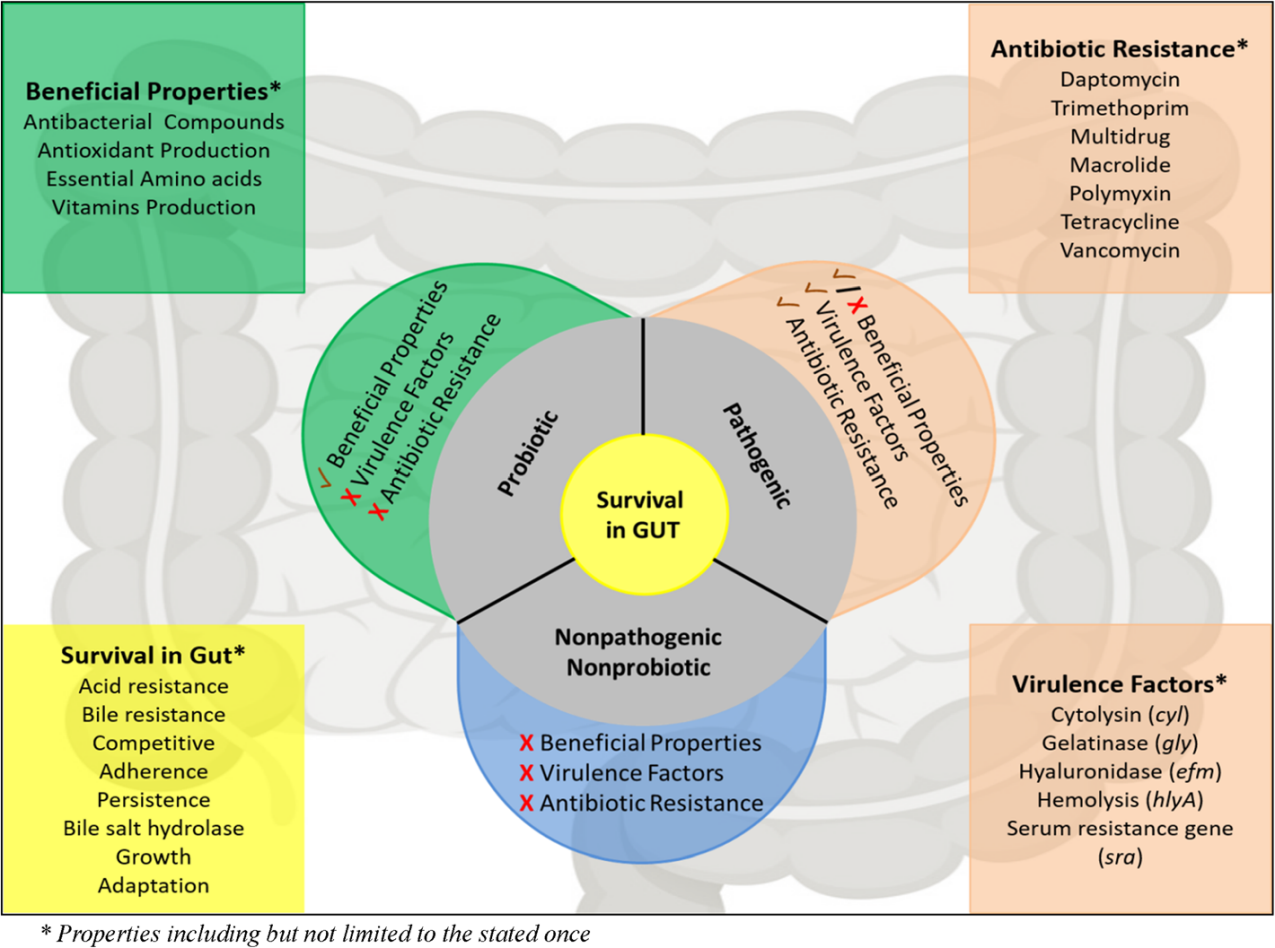
Summarizes properties which can help in delineating probiotic, pathogenic and NPNP strains of *E. faecium*. ✓ indicates presence of a property, X indicates absence of property and ✓ / X indicates either presence or absence of a property

The core genes (1935) were used to construct a phylogenetic tree of the 10 strains along with *Enterococcus faecalis* symbioflor as an out-group. Phylogenetic reconstruction by using Maximum Likelihood method separated 10 strains in 3 distinct clusters with high bootstrap support (bootstrap > 90) (Fig. 7). We found no clustering based on the source of isolation, while strain 17OM39 was closely related to the probiotic strain T110. Similar tree topology was observed for pan genome-based phylogenetic reconstruction (data not shown). Sixty seven genes belonging to antibiotic resistance (22), virulence factors (14) and survival in GIT (31) were used for Principal Component Analysis (PCA) (Additional file 1: Table S6). PCA plot based on euclidean distances showed a distinct grouping of strains based on probiotic, pathogenic and NPNP groups (Fig. 8). The BLAST Atlas was generated with the help of GVIEW server with strain Aus0004 as the reference genome (Fig. 9). Of the strains, Aus0085 exhibited highest relatedness to the reference strain. Significant variable regions were identified among NPNP, pathogenic and probiotic group illustrating their dissimilarity in genomic content. However, several phages and transposon-related loci from the reference strain appeared to be absent in marketed probiotic T110 and 17OM39 strains. This observation further supported their distinct segregation into independent clades. Figure 10 summarizes properties which can help in delineating probiotic, pathogenic and NPNP *E. faecium* strains.

## Discussion

The genus *Enterococcus* is one of the diverse and ecologically significant group, and members of this genus are ubiquitously distributed in nature [1–7]. Numerous studies in the last decade have validated the safety claim of enterococci in foods and as probiotics [15–18]. The diversity and genomic plasticity of *E. faecium* is accountable for both probiotic and pathogenic nature [28–30]. In this study, we have carried out comparative genome analysis to identify genes/pathways which can help in distinguishing probiotic, pathogenic and NPNP strains of *E. faecium.* Further, we have also tried to describe the genetic differences between strain 17OM39 with marketed probiotic, non-pathogenic non-probiotic (NPNP), and pathogenic strains.

The study was carried out on 10 strains, and these strains were grouped into probiotic, non-pathogenic non-probiotic (NPNP) and pathogenic based on the literature survey (Table 1). We could not observe any correlation between higher G + C content with higher number of coding sequences as described in the earlier study [33]. On further annotation by RAST we identify enriched carbohydrates metabolism in all the strains and this is in agreement with the *E. faecium* ability to utilize a wide range of mono-, di-, oligo-saccharides [34, 35]. The comparative genome analysis revealed proportion of core (23.95%), accessory (70.80%) and unique (5.25%) genes. Also, the pan-genome size grew continuously with addition of strains indicating an open pan-genome while size of the core-genome gradually stabilized. These results are in accordance with the previous study for *E. faecium* genome [36]. The small size of core genome and huge number of accessory genes support the observation of the genomic fluidity in *E. faecium* [37].

Functional analysis of core genes have shown that 11.30% of genes were ascribed to carbohydrate metabolism which is in agreement with an earlier report [38] and the distribution of genes in categories of secondary metabolism and motility as contrast to earlier reports [38, 39]. Functional analysis of the accessory genes showed two important subsystems viz. a. carbohydrate metabolism and replication, and b. recombination and repair system. The carbohydrate metabolism was abundant in probiotic group while later in the pathogenic group (Fig. 3), this can be been attributed to the properties of probiotic strains to utilize various carbohydrates [31], while the pathogenic group had higher abundance of replication and recombination genes known to be associated with a large number of mobile elements [1, 2]. Comparison of accessory genes between probiotic and pathogenic group helped in identifying two manganese-containing catalase gene, which provide resistance to hydrogen peroxide present in human GIT [40–42]. Among unique genes, Phosphotransferase (PTS) system for mannose/fructose/sorbose was present in probiotic strain 17OM39 which is involved in sugar uptake [43, 44] while marketed probiotic strain T110 had macrolide-efflux transmembrane protein responsible for drug efflux pump which plays a key role in drug resistance [45, 46]. Moreover, large percentages (61.19%) of unique genes were assigned to an uncharacterized group. Further studies will be required to examine the unexplored attributes.

Enterococci can exhibit resistance to a number of antibiotics, which have been attributed to their innate resistance and ability to successfully acquire resistance through horizontal gene transfer (HGT) [47, 48]. Multiple-drug-resistant strains of *E. faecium* have been increasingly associated with nosocomial infections particularly the vancomycin resistance [49]. Genes conferring resistance to kanamycin were found across all the genomes as this has been attributed to intrinsic property within *E. faecium* [50]. In our study, genes imparting resistance to one or more antibiotics were seen in different strains of *E. faecium* (Table 2). Overall, the pathogenic group of *E. faecium* was found to have higher prevalence of antibiotic resistance genes; a factor that contributes to the challenge of selecting therapeutic measures. The probiotic group was devoid of any major clinically relevant antibiotic resistance [9, 31].

Virulence genes contribute to the pathogenicity of an organism [51]. Despite the increasing knowledge of *E. faecium* as an opportunistic pathogen, the distribution of virulence factors is still poorly understood [51]. Knowledge of the virulence characteristics helps to understand the complex pathogenic process of the pathogenic strains. The ability to adhere to the GIT is reflected to be one of the main selection criteria for potential probiotics as it extends their persistence in the intestine [52] and thus allows the bacterium to exert its probiotic effects for an extended time. However, adhesion is also considered a potential virulence factor for pathogenic bacteria [53]. The intestinal mucus is an important site for bacterial adhesion and colonization [54], and thus adherence property is beneficial to humans in case of probiotics, and it possesses adverse effects in pathogenic strains. The genes described as adherence factors (*acm, scm, Ebp*A*, Ebp*C) in *Enterococcus* have been attributed to pathogenic group. All pathogenic strains showed presence of enterococcus surface protein (*esp)* gene which contributes as a major virulence factor, except strain DO [55–58]. The operon for *bop*D gene involved in biofilm was intact in pathogenic group, but was absent in probiotic and NPNP groups [9]. Nonsense mutation was seen in hyaluronidase and *acm* gene in both probiotic and NPNP strains suggesting its non-functionality. Also, virulence genes *scm, efa*A and *srt*C are not well characterized as virulence determinants in *E. faecium* [9] (Table 3). Although, one could expect a virulence trait depending on the source of isolation, our study did not find any such traits and differed from the earlier reports [59]. Also, the strains showed significantly different patterns of virulence determinants, which underlines the findings of another author [59]. The strain 17OM39 within the probiotic group was devoid of any clinically relevant functional virulence determinants.

MGEs play an important role in the HGT [38, 60–62]. A number of MGEs have been described in *E. faecium* including transposons, plasmids, and bacteriophages [32]. ISs are possibly the smallest and most independent transposable elements which play an important role in shaping the bacterial genomes [63]. IS1542 was present only in probiotic group, and earlier studies on IS1542 have shown its presence in just 2 out of 65 human pathogenic strains, suggesting no direct relation with the strains pathogenicity [64]. The presence of the insertion sequence families in all groups imply that these elements may spread by HGT [36]. However, particular IS elements were distributed in only one group suggesting that these IS elements might have evolved over the time [36, 65]. Notably, the presence of IS16 has been used as a marker within the hospital strains of *E. faecium* with 98% sensitivity and 100% specificity [36]. This observation was further supported by the detection of IS16 in only pathogenic group of *E. faecium* strains. Moreover, ISEfa11 and ISEfa5 were found to be associated with vancomycin resistance genes viz. *VanS, VanX,* and *VanY* [36, 66]. A strong correlation between the IS elements and virulence factors was observed in all genomes (Fig. 4) suggesting that these IS elements might transfer virulence factors. Further studies are required to verify these findings.

*E. faecium* are known to harbour bacteriophages, hence the presence of prophage was predicated in all the ten genomes [36, 67]. Bacteriophages contribute actively to bacterial evolution by integrating and excising from the genome [67]. In certain conditions, they provide new genetic properties to the bacterial host leading to the development of new pathogens, as shown for *Escherichia coli*, *Vibrio cholera* and *Corynebacterium diphtheriae* [68–70]. Various prophages were identified within the strains under study (Additional file 1: Table S2). We could not find any known functional virulence factors or genes associated with probiotic or pathogenic properties within these bacteriophage regions. Further, CRISPRs system was found to be absent within the genomes as oppose to its closest neighbour, *E. faecalis* [71].

Genomic islands are distinct DNA fragments differing between closely related strains, which usually are associated with mobility [72, 73]. The choloylglycine hydrolase gene was found to be present in the genomic island of probiotic strain T110, pathogenic strain 6E6 and NPNP strain 64/3. This gene imparts resistance to bile salts and thus help in survival within the gut environment [74, 75]. A higher similarity was observed between genomic islands of probiotic and NPNP strains as compared to pathogenic group (Fig. 5). The underlying mechanisms for probiotic, NPNP and pathogenic properties by *E. faecium* may be intrinsic or acquired by horizontal exchange of genetic material. Genes found within genomic islands can be considered as acquired properties [14, 72, 73, 76, 77] while others as intrinsic. As all the genes/pathways associated with probiotic properties were not found within MGEs for strain 17OM39, they can be considered as intrinsic. From this study, it is evident that MGEs play an important role in driving the evolution of *E. faecium* strains by adding new genetic features [74, 75]. Certainly, studies like one carried out here will be helpful to understand the evolution of predominant strains.

Biologically active microorganisms are usually required at the target site to induce health benefits or pathogenic effect. To induce such effects it is necessary for the organism to survive and persist in the GIT [78–81]. All the groups showed presence of genes that impart resistance to acid, bile, hydrolyse bile salt and were also able to adhere and grow in the GIT. This finding correlates with the fact that *E. faecium* are normal inhabitants of the gut [82] (Table 4). We found Permease IIC component gene accountable for catalysing the phosphorylation of incoming sugar substrates which helps in competence and survival [83] only in the probiotic group.

Along with the survival ability in GIT, a probiotic strain should be capable of producing antimicrobial substances but on the other hand, should be devoid of acquired antibiotic resistance [84–87]. Furthermore, they must give beneficial effects to the host by producing essential amino acids and vitamins. For strains 17OM39 and T110 (marketed probiotic), we could trace complete pathways for amino acid synthesis viz. valine, lysine, and methionine (Table 5). These are among the essential amino acids and need to be supplied exogenously to humans [88]. Vitamins such as folate and thiamine are the components of Vitamin-B and are considered as essential nutrients for humans. Folate (folic acid) cannot be synthesized by human cells and hence is necessary to be supplemented exogenously as it plays an important role in nucleic acid synthesis and amino acid metabolism [89–91]. Thus, strains (T110 and 17OM39) producing such amino acids and vitamins can be considered beneficial for humans [92, 93]. Antibacterial activity (bacteriocin gene) specific against *Listeria* were found were found in probiotic strains. Genes for exopolysaccharide (EPS) and anti-oxidant production (hydro-peroxidases) were also found in the probiotic strains, thus helping them to establish in the GIT. In summary, probiotic strains have pathways/genes imparting beneficial effects to human host unlike NPNP and pathogenic group.

Earlier studies within *E. faecium* isolates have shown the abundance of plasmids by finding 1–7 number of plasmids in 88 out of 93 isolates [94]. Plasmids comprise a substantial portion of the accessory genome and are accountable for antibiotic and virulence properties which are usually acquired by the HGT [94]. Plasmid from marketed probiotic strain T110 showed 66% similarity to the cytolysin (*cyl*) gene, an important determinant in lethality of endocarditis [94]. Various antibiotics resistance genes viz. vancomycin, streptothricin, erythromycin, gentamicin and kanamycin resistance were identified in plasmids of pathogenic group (Additional file 1: Table S4).

Multi-Locus Sequence Analysis (MLSA) based phylogeny using 6 housekeeping genes (*adk*, *atp*A, *gyd*, *gdh*, *ddl*, *pur*K and *pst*S) could not distinguish between pathogenic and non-pathogenic strains of *E. faecium* [95], but this could be achieved by the core genome SNP based phylogeny [96–99]. Thus the phylogenetic reconstruction by using Maximum likelihood method on core genome separated 10 strains in 3 distinct clusters with high bootstrap support (bootstrap > 90) (Fig. 7). Additionally, the PCA plot based on euclidean distances showed a distinct grouping of strains based on probiotic, pathogenic and NPNP groups (Fig. 8). Pathogenic Island (2,812,458-2,878,042 and 1,860,143-1,894,650 bp) were identified in the genome atlas and mainly consist of virulence-associated genes, IS elements, transposes, integrases and antibiotic resistance-related genes. It also has vancomycin resistance gene cluster and presence of *esp* gene which correlates with the previous studies [100, 101].

## Conclusions

This study provides valuable insights based on the genomic differences of probiotic, NPNP and pathogenic strains of *E. faecium*. Analyses of core, accessory and unique genes present in the genomes have helped in differentiating strains with different properties. We observed a strong correlation between insertion sequence elements and virulence factors in pathogenic *E. faecium* strains which needs to be investigated further. Moreover, the analysis of intrinsic and acquired properties helped us to know the inherent probiotic properties of strain 17OM39. The work presented here demonstrates that comparative genomic analyses can be applied to large numbers of genomes, to find potential probiotic candidates.

## Methods

### Bacterial sequences and strains

Whole Genome Sequence of *E. faecium* was retrieved from NCBI genomes, and a total of ten strains were used in this study. The genome for 17OM39 was sequenced using the Illumina MiSeq platform using 2 × 300 paired-end libraries. *De-novo* assembly method was employed to carry out the assembly of quality-filtered reads using MIRA assembler version 4.9.3 [102]. All the genomes were RAST annotated [103].

### Comparative analysis

Comparative analysis of ten whole genome sequences of *Enterococcus faecium* was done by an ultra-fast bacterial pan-genome analysis pipeline (BPGA) [104] which performs GC content analysis, pan-genome profile analysis along with sequence extraction and phylogenetic analysis. Furthermore, the genome was investigated for the presence of putative virulence genes using Virulence Factor of Bacterial Pathogens Database (VFDB) [105]. Screening of probiotic genes was done by performing a BLAST of probiotic genes to the genome by online NCBI’s BLASTX tool [106]. Comprehensive Antibiotic resistance Database (CARD) was used for analysis of antibiotic resistance [49]. Presence of CRISPR repeats was predicted using the CRISPRFinder tools [107]. PHASTER: rapid identification and annotation of prophage sequences within bacterial genomes were used for identification of prophages within the genome [108]. Bacterial insertion elements (ISs) were identified by ISfinder [109]. Horizontal gene transfer was detected by genomic island tool: Islandviewer [110, 111]. The clustering and annotation of protein sequences were done with the help of orthoMCL [112]. COG analysis was done with the help of webMGA server [113]. STAMP software was used to generate a PCA plot [114]. A blast atlas was generated with the help of GVIEW Server (https://server.gview.ca/) [115].

## Additional files

Additional file 1: **Figure S1** Features assigned to subsystems from RAST present in all ten Enterococcus strains. **Figure S2. (A)** Proportion of known, hypothetical and unknown proteins in the group of core, accessory and unique genes **(B)** Venn Diagram for accessory genome between probiotic, non-pathogenic and pathogenic group. **Figure S3.** Functional analysis of the accessory genes in COG categories. **Table S1.** IS elements found in *Enterococcus* genomes by ISfinder tool. + Present, − Absent. **Table S2.** Number of Phage elements present in *Enterococcus* genomes as intact, questionable and incomplete. **Table S3.** Number of Genomic Islands in *Enterococcus* genomes. **Table S4.** Antibiotic Resistance genes found in *Enterococcus* plasmids as performed by CARD analysis, where + Present and - Absent. **Table S5.** IS elements found in *Enterococcus* plasmids by ISfinder tool. + Present, − Absent. **Table S6.** Table showing the various genes used in the study for generating PCA plot. (DOCX 351 kb)

Additional file 2: Detailed information on the core genes present in the genomes under study (XLSX 57 kb)

Additional file 3: Detailed information on the accessory genes present in the strains under study. (XLSX 115 kb)

Additional file 4: Detailed information on the unique genes present in the strains under study. (XLSX 29 kb)

Additional file 5: Detailed information on genes present in genomic islands in all the strains. (XLSX 137 kb)

## Abbreviations

BPGA: Ultra-fast bacterial pan-genome analysis pipeline
CARD: Comprehensive antibiotic resistance database
CDS: Coding DNA sequence
EPS: Exopolysaccharides
GI: Genomic Island
GIT: Gastrointestinal tract
HGT: Horizontal gene transfer
ISs: Insertion sequences
MGEs: Mobile genetic elements
PTS: Phosphotransferase system
VFDB: Virulence Factor of Bacterial Pathogens Database
