# Supplementary material for: Comparative genome analysis reveals key genetic factors associated with probiotic property in Enterococcus faecium strains
Source: BMC Genomics. 2018 Sep 4;19:652. doi: 10.1186/s12864-018-5043-9 (PMC6122445; doi:10.1186/s12864-018-5043-9)
Supplement: Supplementary file 1 — Figure S1 Features assigned to subsystems from RAST present in all ten Enterococcus strains. Figure S2. (A) Proportion of known, hypothetical and unknown proteins in the group of core, accessory and unique genes (B) Venn Diagram for accessory genome between probiotic, non-pathogenic and pathogenic group. Figure S3. Functional analysis of the accessory genes in COG categories. Table S1. IS elements found in Enterococcus genomes by ISfinder tool. + Present, − Absent. Table S2. Number of Phage elements present in Enterococcus genomes as intact, questionable and incomplete. Table S3. Number of Genomic Islands in Enterococcus genomes. Table S4. Antibiotic Resistance genes found in Enterococcus plasmids as performed by CARD analysis, where + Present and - Absent. Table S5. IS elements found in Enterococcus plasmids by ISfinder tool. + Present, − Absent. Table S6. Table showing the various genes used in the study for generating PCA plot. (DOCX 351 kb) [file 12864_2018_5043_MOESM1_ESM.docx]

**SUPPLEMENTARY MATERIAL**

**FIGURES**

**
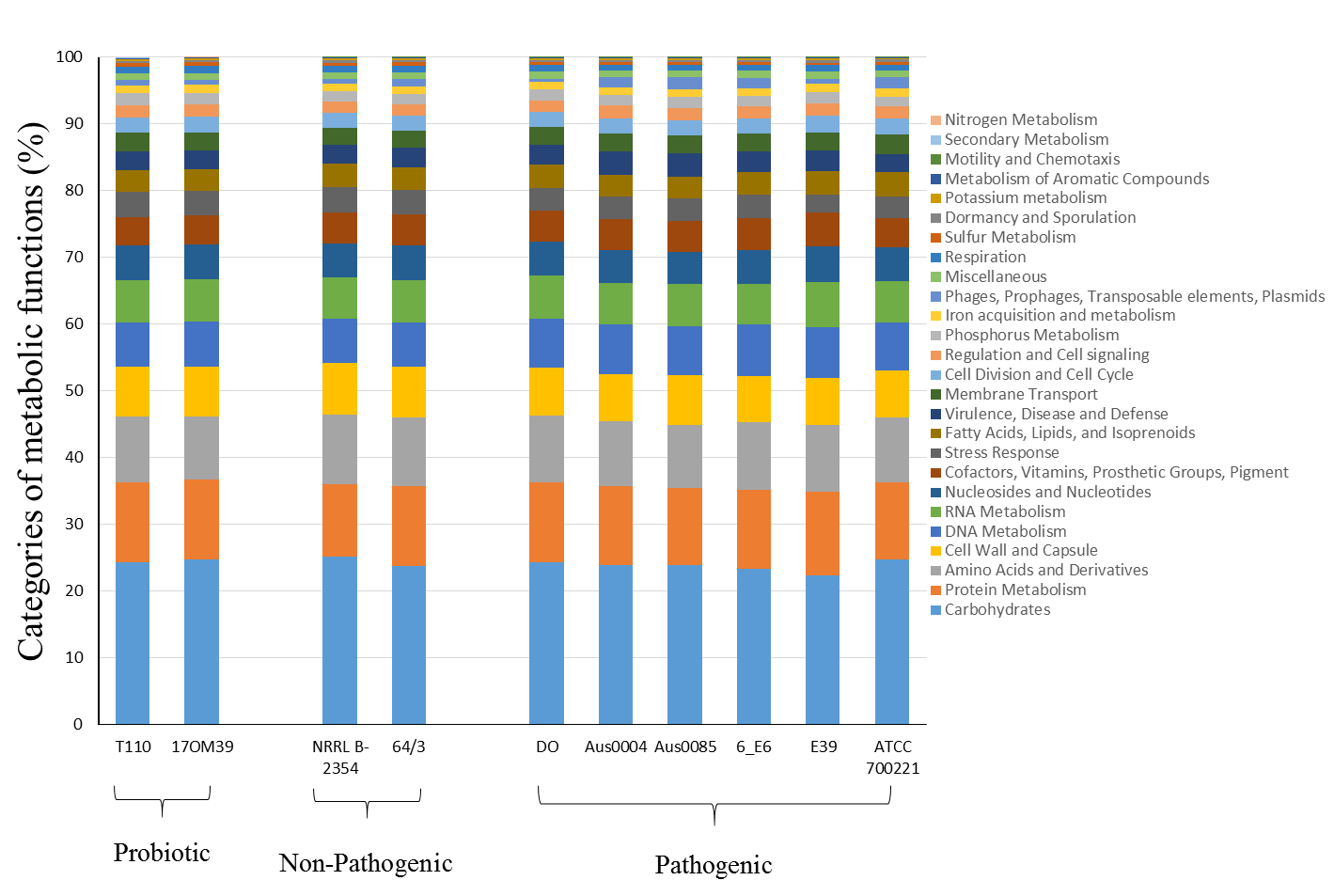
**

**
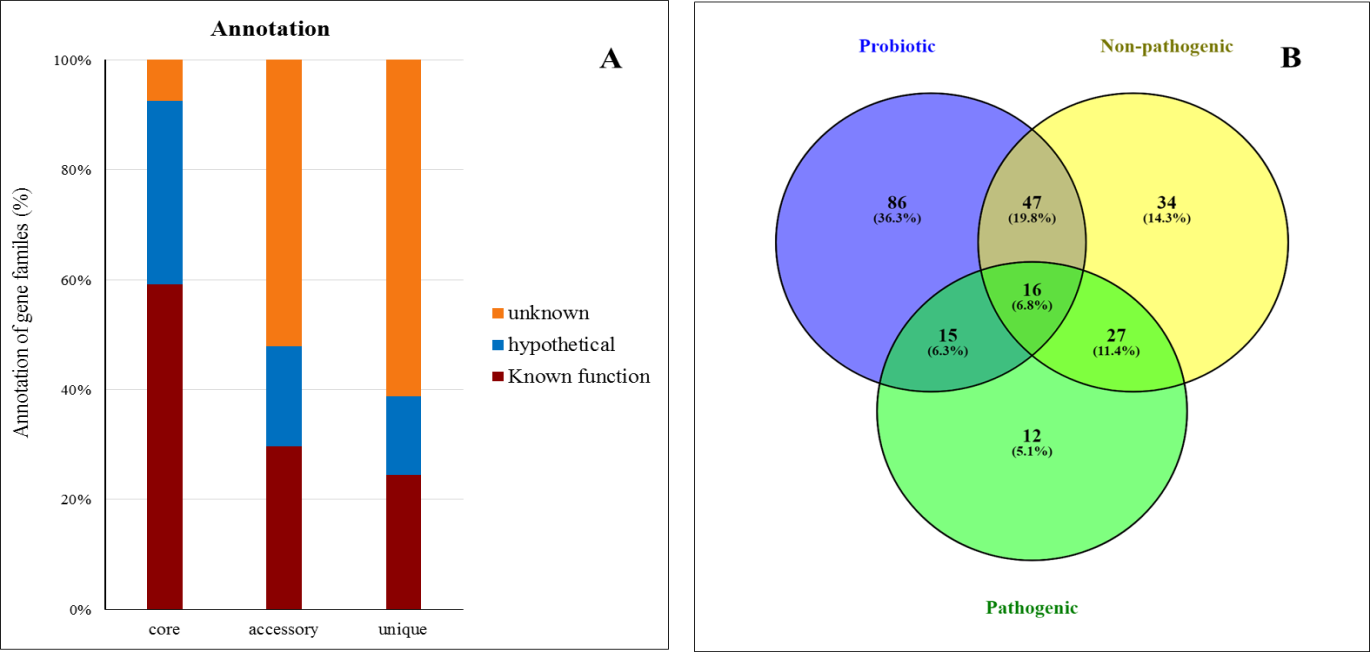
**


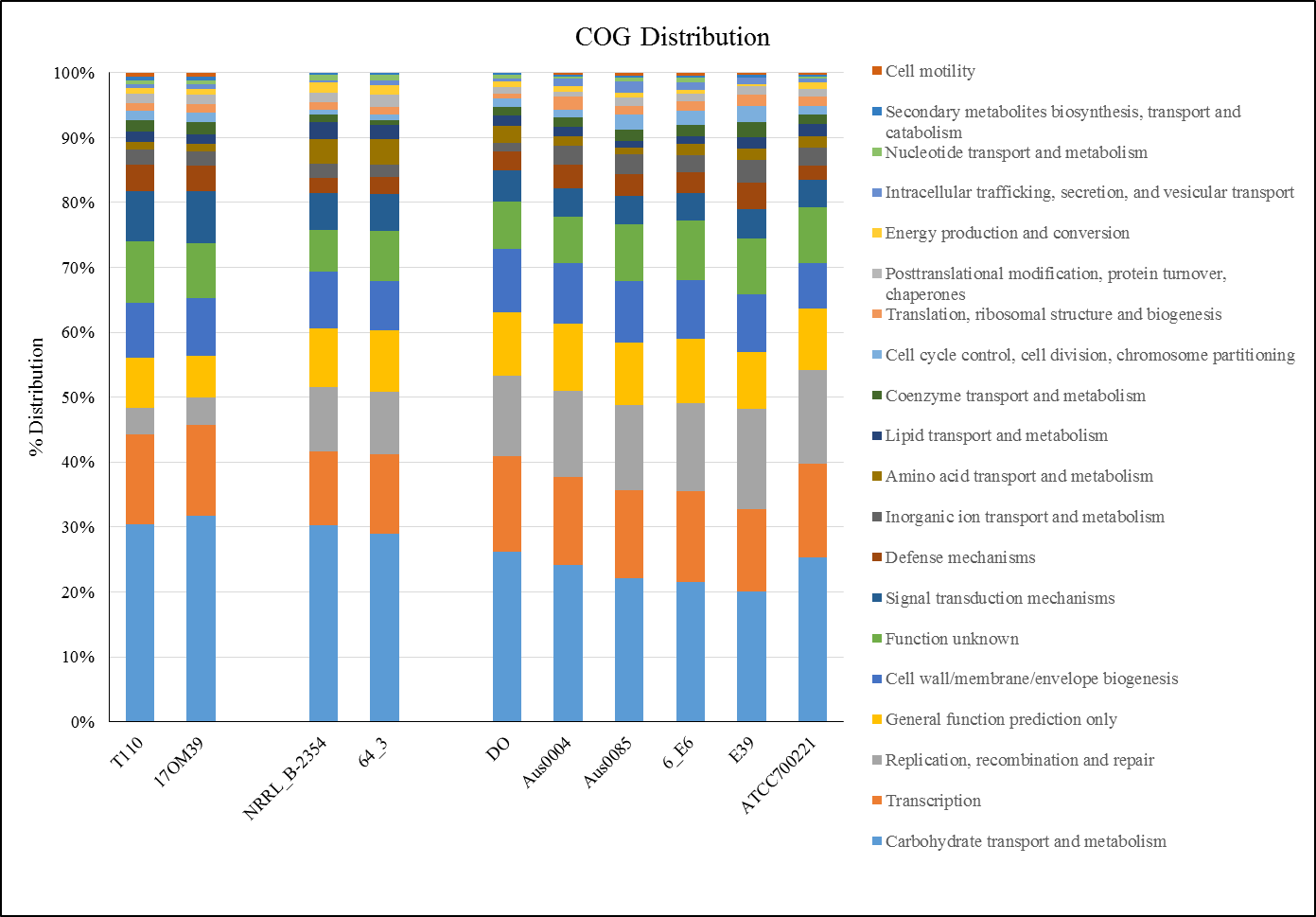


**TABLES**

| **IS elements** | **Probiotic** | **Non-Pathogenic** | **Pathogenic** |
| --- | --- | --- | --- |
| IS1216 | **-** | **+** | **+** |
| IS1216E | **-** | **+** | **+** |
| IS1216V | **-** | **+** | **+** |
| IS1251 | **-** | **+** | **+** |
| IS1476 | **-** | **-** | **+** |
| IS1485 | **+** | **+** | **+** |
| IS1542 | **+** | **-** | **-** |
| IS16 | **-** | **-** | **+** |
| IS1678 | **-** | **-** | **+** |
| IS19 | **+** | **+** | **+** |
| IS256 | **-** | **-** | **+** |
| IS6770 | **-** | **+** | **+** |
| ISEf1 | **-** | **-** | **+** |
| ISEfa10 | **-** | **+** | **+** |
| ISEfa11 | **-** | **-** | **+** |
| ISEfa12 | **-** | **+** | **-** |
| ISEfa13 | **-** | **-** | **+** |
| ISEfa4 | **-** | **-** | **+** |
| ISEfa5 | **-** | **-** | **+** |
| ISEfa7 | **-** | **-** | **+** |
| ISEfa8 | **-** | **-** | **+** |
| ISEfm1 | **+** | **+** | **+** |
| ISEfm2 | **+** | **-** | **+** |
| ISEnfa110 | **-** | **-** | **+** |
| ISEnfa3 | **-** | **-** | **+** |
| ISEnfa4 | **+** | **-** | **+** |
| ISLgar5 | **+** | **-** | **+** |
| ISS1W | **-** | **+** | **+** |
| ISSsu5 | **-** | **-** | **+** |

**Table S1** IS elements found in *Enterococcus* genomes by ISfinder tool. + Present, - Absent

| **Phage** | **17OM39** | **T110** | **NRRL B-2354** | **64/3** | **DO** | **Aus0004** | **Aus0085** | **6_E6** | **E39** | **ATCC 700221** |
| --- | --- | --- | --- | --- | --- | --- | --- | --- | --- | --- |
| Intact | 1 | 2 | 2 | 2 | 1 | 3 | 3 | 3 | 2 | 4 |
| Questionable | 1 | - | - | - | 1 | 1 | 1 | 1 | 2 | 2 |
| Incomplete | - | - | - | - | 1 | 1 | 1 | - | - | - |
| Total Size (bp) | 73100 | 68700 | 87000 | 89100 | 74500 | 164900 | 220600 | 208400 | 117000 | 268300 |

**Table S2** Number of Phage elements present in *Enterococcus* genomes as intact, questionable and incomplete.

| **Genome** | **Genomic Islands** | **Size (bp)** |
| --- | --- | --- |
| 17OM39 | 11 | 172279 |
| T110 | 9 | 117774 |
| NRRL B-2354 | 12 | 142123 |
| 64/3 | 7 | 78409 |
| DO | 7 | 73539 |
| Aus0004 | 35 | 496552 |
| Aus0085 | 24 | 304164 |
| 6_E6 | 8 | 69233 |
| E39 | 17 | 191228 |
| ATCC 700221 | 10 | 119456 |

**Table S3** Number of Genomic Islands in *Enterococcus* genomes

| **Genes** | **6_E6** | **ATCC_700221** | **Aus0085** | **DO** | **E39** |
| --- | --- | --- | --- | --- | --- |
| **Aminoglycoside** | **+** | **+** | **+** | **+** | **+** |
| **Chloramphenicol** | **-** | **-** | **-** | **+** | **-** |
| **Dihydrofolate** | **-** | **+** | **-** | **-** | **-** |
| **Erythromycin** | **+** | **+** | **+** | **+** | **+** |
| **Gentamicin** | **+** | **+** | **-** | **-** | **-** |
| **Lincosamide** | **-** | **-** | **+** | **-** | **-** |
| **Streptothricin** | **-** | **+** | **-** | **+** | **+** |
| **Tetracycline** | **-** | **-** | **-** | **+** | **-** |
| **Vancomycin** | **+** | **+** | **-** | **-** | **+** |

**Table S4** Antibiotic Resistance genes found in *Enterococcus* plasmids as performed by CARD analysis, where + Present and - Absent

| IS elements | 6_E6 | ATCC 700221 | Aus 0004 | Aus 0085 | DO | E39 | NRRLB 2354 | T110 |
| --- | --- | --- | --- | --- | --- | --- | --- | --- |
| IS1062 | - | - | - | - | + | - | - | - |
| IS1182 | - | + | - | - | + | + | - | - |
| IS1216 | + | + | - | + | + | + | + | - |
| IS1216E | + | + | - | + | + | + | + | - |
| IS1216V | + | + | - | + | + | + | + | - |
| IS1251 | - | + | - | - | - | + | + | - |
| IS1297 | + | - | - | + | + | + | + | - |
| IS1476 | - | + | - | - | + | - | - | - |
| IS1485 | + | + | - | + | + | + | + | - |
| IS16 | + | - | - | - | + | - | - | - |
| IS19 | + | + | - | + | + | + | + | - |
| IS256 | + | + | - | + | - | + | + | - |
| IS6770 | - | + | - | - | + | - | - | - |
| ISCco2 | + | + | - | + | + | + | - | - |
| ISEf1 | + | + | - | + | + | + | + | - |
| ISEfa10 | + | + | - | - | - | + | - | - |
| ISEfa11 | + | + | - | - | + | + | + | - |
| ISEfa12 | - | - | - | - | + | - | - | - |
| ISEfa13 | + | - | - | - | - | - | - | - |
| ISEfa4 | + | - | + | + | + | + | - | - |
| ISEfa5 | + | + | - | - | + | + | + | - |
| ISEfa7 | + | + | - | + | + | + | - | - |
| ISEfa8 | + | + | - | + | + | + | - | - |
| ISEfm1 | + | + | - | + | + | + | + | - |
| ISEfm2 | + | + | - | - | + | + | - | - |
| ISEnfa3 | - | - | - | + | - | - | - | - |
| ISEnfa4 | + | + | - | + | - | + | + | - |
| ISLgar5 | + | - | - | + | - | - | - | - |
| ISLpl1 | - | - | - | - | - | - | + | - |
| ISPp1 | - | - | - | - | - | - | + | - |
| ISS1CH | + | - | - | + | + | + | + | - |
| ISS1D | + | - | - | + | + | + | + | - |
| ISS1E | + | - | - | + | + | + | + | - |
| ISS1M | + | - | - | + | + | + | + | - |
| ISS1N | + | - | - | + | + | + | + | - |
| ISS1W | + | + | - | + | + | + | + | - |

**Table S5** IS elements found in *Enterococcus* plasmids by ISfinder tool. + Present, - Absent

|  | Properties | Probiotic | | NPNP | | Pathogenic | | | | | |
| --- | --- | --- | --- | --- | --- | --- | --- | --- | --- | --- | --- |
| Catergories | Genes | 17OM39 | T110 | 64_3 | NRRL B  2354 | 6E_6 | ATCC  700221 | Aus  0004 | Aus  0085 | DO | E39 |
| Survival in Gut | abpT | + | + | + | + | + | + | + | + | + | + |
|  | bsh | + | + | + | + | + | + | + | + | + | + |
|  | LBA1272 | + | + | + | + | + | + | + | + | + | + |
|  | clpC | + | + | + | + | + | + | + | + | + | + |
|  | clpE | + | + | + | + | + | + | + | + | + | + |
|  | copA | + | + | + | + | + | + | + | + | + | + |
|  | dltA | + | + | + | + | + | + | + | + | + | + |
|  | dltB | + | + | + | + | + | + | + | + | + | + |
|  | dps | + | + | + | + | + | + | + | + | + | + |
|  | met | + | + | + | + | + | + | + | + | + | + |
|  | cdpA | + | + | + | + | + | + | + | + | + | + |
|  | gadC | - | - | - | - | + | - | - | + | + | + |
|  | Lr1584 | - | - | - | - | + | + | + | + | - | + |
|  | LBA0995 | + | + | + | + | + | + | + | + | + | + |
|  | pts14C | + | + | - | - | - | - | - | - | - | - |
|  | FbpA | + | + | + | + | + | + | + | + | + | + |
|  | LJ1656 | + | + | + | + | + | + | + | + | + | + |
|  | LBA1430 | + | + | + | + | + | + | + | + | + | + |
|  | LBA1429 | + | + | + | + | + | + | + | + | + | + |
|  | ispA | + | + | + | + | + | + | + | + | + | + |
|  | lsp | - | - | + | + | + | + | + | + | + | + |
|  | LBA0867 | + | + | + | + | + | + | + | + | + | + |
|  | msrB | + | + | + | + | + | + | + | + | + | + |
|  | gtfA | + | + | + | + | + | + | + | + | + | + |
|  | LBA1524 | + | + | + | + | + | + | + | + | + | + |
|  | Lr1265 | + | + | + | + | + | + | + | + | + | + |
|  | LJ1654 | + | + | + | + | + | + | + | + | + | + |
|  | srtA | + | + | + | + | + | + | + | + | + | + |
|  | treC | + | + | + | + | + | + | + | + | + | + |
|  | rrp1 | - | - | - | - | - | - | + | + | - | - |
|  | xylA | + | + | - | - | - | - | - | - | - | - |
| Virulance Factors | acm | + | + | + | + | + | + | + | + | + | + |
|  | bopD | + | + | + | + | + | + | + | + | + | + |
|  | cpsA | + | + | + | + | + | + | + | + | + | + |
|  | cpsB | + | + | + | + | + | + | + | + | + | + |
|  | cpsJ | + | + | + | + | + | + | + | + | + | + |
|  | EbpA | - | + | + | - | + | + | + | + | + | + |
|  | EbpC | - | + | + | - | + | + | + | + | + | + |
|  | EcbA | - | - | - | - | + | + | + | + | + | + |
|  | EF0818 | + | - | - | + | - | - | - | - | - | - |
|  | efaA | + | + | + | + | + | + | + | + | + | + |
|  | Esp | - | - | - | - | + | + | + | + | - | + |
|  | Scm | + | + | + | + | + | + | + | + | + | + |
|  | SgrA | - | - | - | - | + | + | + | + | + | + |
|  | srtC | + | + | + | + | + | + | + | + | + | + |
| Antibiotic Resitance | AAC | + | + | + | + | + | + | + | + | + | + |
|  | adeC | + | + | + | + | + | + | + | + | + | + |
|  | daptomycin | - | - | - | - | - | - | - | - | - | + |
|  | dfrE | + | + | + | + | + | + | + | + | + | + |
|  | dfrF | - | - | - | - | + | - | - | + | - | + |
|  | dfrG | - | - | - | - | - | - | + | - | - | - |
|  | efmA | + | + | + | + | + | + | + | + | + | + |
|  | efrA | + | + | + | + | + | + | + | + | + | + |
|  | efrB | + | + | + | + | + | + | + | + | + | + |
|  | ErmG | - | - | - | - | - | - | + | - | - | - |
|  | lsaA | + | + | + | + | + | - | + | + | + | + |
|  | mefA | - | - | - | - | - | - | + | - | - | - |
|  | msrC | + | + | + | + | + | + | + | + | + | + |
|  | PmrE | - | - | - | - | - | + | - | - | - | - |
|  | tetL | - | - | - | - | - | - | - | - | - | + |
|  | tetM | - | - | - | - | + | - | + | + | - | + |
|  | vanB | - | - | - | - | - | - | + | + | - | - |
|  | vanHB | - | - | - | - | - | - | + | + | - | - |
|  | vanRB | - | - | - | - | - | - | + | + | - | - |
|  | vanSB | - | - | - | - | - | - | + | + | - | - |
|  | vanWB | - | - | - | - | - | - | + | + | - | - |
|  | vanYB | - | - | - | - | - | - | + | + | - | - |

**Table S6** Table showing the various genes used in the study for generating PCA plot
